# Supplementary material for: Splenic Tropism and Spleen‐Modulated Systemic Inflammation in Acute Plasmodium vivax Malaria
Source: Am J Hematol. 2026 Sep 10;101(12):2976–89. doi: 10.1002/ajh.70498 (PMC13742366; doi:10.1002/ajh.70498)
Supplement: Supplementary file 1 [file AJH-101-2976-s001.docx]

**Supplementary Table 1.** Clinical and laboratory characteristics of acute vivax malaria patient with histopathology data

| **Demographic data** | Age | 17 years |
| --- | --- | --- |
|  | Sex assigned at birth | Male |
|  | Ethnicity | Papuan |
|  | Weight | 111 kg |
|  | Height | 158 cm |
|  | | |
| **Clinical** | Splenectomy | Spleen rupture grade V (trauma: traffic accident) |
|  | Spleen weight | 446 gr |
|  | Fever | Yes |
|  | Symptom history | Headache and nauseous in last 2 days |
|  | Malaria history | 1 *P. vivax* episode in the past year |
|  | Total-body blood volume | 5.62 L |
|  | | |
| **Laboratory** | Haemoglobin | 11.4 g/dL |
|  | White cell count | 23.9 x10^3^/µL |
|  | Platelet count | 185 x10^3^/µL |
|  | Spleen white pulp area | 10.7% |
|  | Spleen red pulp area | 86.2% |
|  | | |
| **Parasitology** | PfHRP2/Pan RDT | Negative |
|  | Plasmodium PCR | *P. vivax* monoinfection |
|  | Peripheral parasitaemia | 0.005 (% red cells); 238 parasites per µL blood |
|  | Splenic parasitaemia | 2.85 (% red cells) |
|  | Total peripheral biomass | 1.34 x10^9^ parasites |
|  | Total splenic biomass | 14.3 x10^9^ parasites |

Footnotes:

Abbreviations: PfHRP2, *Plasmodium falciparum* histidine-rich protein-2; RDT, rapid diagnostic test; PCR, polymerase chain reaction.

**Supplementary Table 2.** Baseline characteristics of spleen-intact patients from cohort 2 without artefactual haemolysis at venesection.

|  | **Spleen-intact**  **(cohort 2)**  **n=36** | **Splenectomized**  **n=25** | **P-value*** |
| --- | --- | --- | --- |
| Age in years  (median [IQR]) | 24 (19.5-31.5) | 24 (18-35.5) | - |
| Sex assigned at birth  (n of males, [%]) | 23 (64) | 21 (84) | - |
| Ethnicity  (n of Papuans, [%]) | 34 (94) | 10 (40) | **-** |
| Body temperature in °C  (median [IQR]) | 37.0 (36.4-37.9) | 37.1 (36.5-38.5) | - |
| Fever days (median [IQR]) | 4 (3-7) | 2 (2-3.5) | **-** |
| Cough  (n [%]) | 11 (31) | 6 (24) | - |
| Respiratory rate per min  (median [IQR]) | 20 (19-24) | 22 (20-24) | 0.008 |
| Haemoglobin in g/dL  (median [IQR]) | 11.9 (10.5-13.1) | 13.6 (12.4-14.3) | 0.0001 |
| Haematocrit in %  (median [IQR]) | 34.4 (28.8-37.0) | 42.1 (39.2-45.6) | <0.0001 |
| Circulating red cells x10^6^/µL  (median [IQR]) | 4.5 (4.0-4.8) | 5.4 (5-5.9) | <0.0001 |
| Circulating white cells x10^3^/µL  (median [IQR]) | 5.5 (4.9-7.6) | 8.6 (6.5-11.6) | 0.0001 |
| Circulating platelets x10^3^/µL  (median [IQR]) | 73 (53-107) | 274 (173-430) | <0.0001 |
| Circulating parasites per µL blood  (median [IQR]) | 6,680  (2,280-11,900) | 21,100  (13,700-45,600) | 0.001 |

Footnotes:

*Mann-Whitney U test used for statistical comparison

Abbreviations: IQR, interquartile range

**Untreated patients with PCR-confirmed acute uncomplicated vivax malaria**

n=50 from Timika, Papua, Indonesia

**Histology case,**

n=1, emergency splenectomy following road traffic accident

***Analysis:*** histology, peripheral blood and spleen parasitaemia and parasite biomass

**Splenectomized Group**

n=25

**Primary spleen-intact Group**

Cohort 1, n=24

***Analysis:*** demographic and clinical characteristics, haematology, parasitaemia, PCR and plasma levels of PvLDH, IL-6, TNF-alpha, IL-1b, IL-18, IL-10, ang-2, NE

***Analysis*** of cell-free haemoglobin

**Historical Spleen-intact Group**

Cohort 2, n=36

**Supplementary Figure 1. Study design and participant cohorts.** The primary analyses compared 24 spleen-intact and 25 previously splenectomized patients with PCR-confirmed acute uncomplicated *Plasmodium vivax* malaria. These cohorts contributed to the clinical, parasitological, PvLDH and inflammatory biomarker analyses. Cell-free haemoglobin measurements were compared with a separate historical cohort of 36 spleen-intact patients due to artefactual haemolysis during venepuncture in the primary spleen-intact cohort. Direct splenic histology and parasite biomass quantification were performed in one additional untreated patient who underwent emergency splenectomy following trauma-related splenic injury prior to antimalarial treatment. Abbreviations: PCR, polymerase chain reaction; PvLDH, *P. vivax* lactate dehydrogenase; IL-6, interleukin-6; TNF-alpha, tumour necrosis factor-alpha; IL-1b, interleukin-1 beta; IL-18, interleukin-18; IL-10, interleukin-10; ang-2, angiopoietin-2; NE, neutrophil elastase.

**Supplementary Figure 2. Correlation between plasma PvLDH and circulating parasitaemia.** Plasma PvLDH concentrations (ng/mL) and circulating parasitaemia (parasites per µL blood) in spleen-intact (n=24) and splenectomized patients (n=25) with uncomplicated vivax malaria were log transformed and correlated using the Pearson test. Linear curves were generated by simple linear regression. Abbreviations: PvLDH, *P. vivax* lactate dehydrogenase.

**Supplementary Figure 3. Neutrophil data.** Comparison of A) circulating neutrophil counts, B) plasma NE concentrations and C) NE-to-neutrophil count ratio in spleen-intact (spleen, cohort 1) and splenectomized individuals (No spleen) with acute uncomplicated vivax malaria in Timika, Indonesia. Data points are individual patients with median and interquartile range shown. Neutrophil counts were available for 24 spleen-intact and 24 splenectomized patients. Plasma NE concentration was used as a marker of systemic neutrophil activation and were available for 20 spleen-intact and 25 splenectomized patients (NE measurements in 4 spleen-intact patients were above the upper limit of detection of the assay and were excluded). The Mann-Whitney U test was used for statistical comparison. Abbreviations: NE, neutrophil elastase.
